# Design and implementation of DATA logging and stabilization system for a UAV

**DOI:** 10.1016/j.heliyon.2024.e26394

**Published:** 2024-02-14

**Authors:** Ganesh Kumar Siva Sivamani, Abhishek Gudipalli

**Affiliations:** School of Electrical Engineering, Vellore Institute of Technology, Vellore, Tamilnadu, India

**Keywords:** Data logging, MEMS sensors, Arduino, Flight data

## Abstract

In this paper integration of Micro Electro Mechanical Systems (MEMS) based Inertial Measurement Unit (IMU) and Global positioning System are used for data logging. This paper presents the design and implementation of a data logging system for an Unmanned Aerial Vehicle. The main focus is designing a prototype system for tracking purposes with image-capturing capabilities. The purpose is to develop a recorder that can provide a complete record of the parameters in case of surveillance. The data logger can also be used to determine the cause of a flight crash. The results are gathered to improve the quality of measurements. And also the quality of the sensor's measurements can be improved with sophisticated filtering techniques. This filter was designed using the Mahony filter which provides efficient and effective solutions for IMU. This filter is computationally efficient and requires fewer mathematical computations to produce desired results at lower sampling rates. Additionally, the pitch, roll, and yaw stabilization of the device must be done when the system enters abnormal conditions. However, altitude, camera angle, and motion blur make it a more challenging task.

## 1 Introduction

At present, the data acquisition system is regarded as an essential component of the aircraft's instrumentation. It is a tool for gathering data or information from the target region. In the aviation business, it is frequently used to gather all crucial flight data in real-time. The information collected is either broadcast to the ground station from the onboard system or stored in the aircraft's storage device, which can be depleted. The real-time data comprises critical technical information such as control surface deflections, airspeed, altitude, air density, engine thrust, absolute pressure, engine RPM, and other necessary factors. In addition, it records non-technical data like cockpit voices, which can be helpful for post-flight analysis.

The author [1] has applied low-cost sensors for the Guidance, Navigation, and Control (GNC) of an autonomous Unmanned Aerial Vehicle (UAV). The real-time findings of using a low-cost Inertial Measurement Unit (IMU) and Global Positioning System (GPS) receiver for the GNC are presented in this study. For autonomous flight, the INS/GPS navigation loop offers continuous and dependable navigation solutions to the guidance and flight control loop. UAVs were initially developed for military purposes and are now finding increasing application in the civilian sector. China's unmanned aerial vehicle (UAV) business did not get its start until the 21st century, and it has only just begun a period of tremendous growth. UAVs have recently emerged as a viable option for remote sensing due to their portability, versatility in take-off and landing, lack of positional and temporal constraints, and rapid area coverage [5].

A new technique for calibrating a low-cost six-degree-of-freedom MEMS inertial Navigation system for use in an unmanned aerial vehicle (UAV). Inter-axis misalignment correction is used to simulate the accelerometer and gyroscope. At least nine equations must be solved to establish the calibration parameters of a tri-axis accelerometer (3 scale factor, 3 zero bias, 3 misalignment angles) [2]. Any abnormalities arise in any sensor parameter immediately after the data acquisition system starts recording and transmitting the signal to the base station or recorded in the SD module for further analysis [3].

Unmanned aerial vehicle (UAV) sensors and platforms are currently employed in practically every industry (for example, agriculture, forestry, and mining) that requires information viewed from the top or oblique perspectives. While they promise to be a generic remote sensing instrument, the necessary Remote sensing data processing and analysis methods [4]. The author [6] has evaluated to interface integrity and sensor performance, the Autopilot hardware was installed and flown in NAL-Slybird. The experiment was carried out in Bangalore, where the altitude above sea level is around 900 m. A number of flight tests were undertaken, and a large amount of data was captured utilizing the onboard microSD memory as a Modular Data Logger.

Due to recent developments in MEMS (Micro-electromechanical Systems), a hybrid IMU sensor array composed of a 3-axis accelerometer, a 3-axis gyroscope, and a tri-axis magnetometer is now possible; hence the name “MAGG” sensor. This sensor can take accurate orientation readings concerning both the direction of gravity and the direction of the Earth's magnetic field [7]. There is a risk of data corruption or loss if the platform flies too far outside the range of the radio transmission. Therefore, the system should have an additional in-built logging capability for critical status variables. This function is independent of radio connection quality issues and enables the diagnosis of system failure. It's a “black box” in the lingo of traditional aviation. A quadcopter is an Unmanned Aerial Vehicle (UAV) with four driving motors. It is extensively utilized in a variety of industries, including agricultural, due to its basic design and simple motion idea [8].

Unmanned aerial vehicles (UAV) have been used in a wide variety of military and civilian endeavours, from target acquisition and surveillance missions to agricultural spraying. Research into unmanned aerial vehicles (UAVs) has recently seen a surge in popularity thanks to the falling prices and rising efficiency of key components including batteries, electric motors, and microcontrollers [9]. Wearable magnetic and inertial measuring units that are lightweight and low-cost have found several applications, such as aerial aircraft navigation or human motion analysis, where 3D orientation tracking of a rigid body is of interest. However, due to flaws in gyroscope, accelerometer, and/or magnetometer data within a magnetic and inertial measuring unit, various research have suggested sensor fusion algorithms to reliably and robustly predict the 3D orientation [10].

More sensors can be used for data acquisition, and the mobility of UAVs allows for more varied approaches to data collection. The use of UAVs has many benefits in this context, including the capacity to collect overlapping photographs from different viewing angles and the ability to approach close to an object due to the UAV's great maneuverability in urban situations [11].

The need for autonomous and more mechanized procedures, that eliminate human error, is growing as the world grows more dependent on technology. To be effective, safe, and undetectable during visual condition assessments in locations inaccessible to people, equipment utilized for this purpose must be compact and silent [12].

The author in Ref. [30] has performed by designing a cascade proportional integral derivative (PID) control strategy that deals with the disturbances. This paper uses the extended Kalman filter. Since extended Kalman filter requires a lot of computations and hence is complex to implement. In comparison with the above method my paper uses the Mahony filter so that the mathematical calculations are less and also the execution time is less.

The author of the paper [31] wants to improve the long-term performance of traditional SINS/GPS navigation systems by employing a fuzzy adaptive integration technique. The primary idea underlying the proposed adaptive integration is that the attitude-heading reference system (AHRS) performs well in low-accelerated movements but degrades in manipulated or accelerated motions.

In paper [32] aerial, ground, and marine vehicles, assisted microelectromechanical system (MEMS), inertial navigation system (INS) and global navigation satellite system (GNSS) are two frequent sources of location and velocity information.

GNSS systems may offer precise information on absolute three-dimensional location and even velocity. Furthermore, it is quite stable and can maintain a stable level of navigation process for an extended period.

However, GNSS performance is affected by the external environment and satellite accessibility. For error correction, the Kalman filter and neural network are used. The author describes a new immersion and invariance (I&I) observer for inertial microelectromechanical systems (MEMS) sensors-based, low-cost attitude-heading reference systems. Immersion and invariance (I&I) is a control and observation strategy that uses system immersion and manifold invariance to achieve design goals [33].

## 2 Proposed methodology and discussion

**Fig-1:**
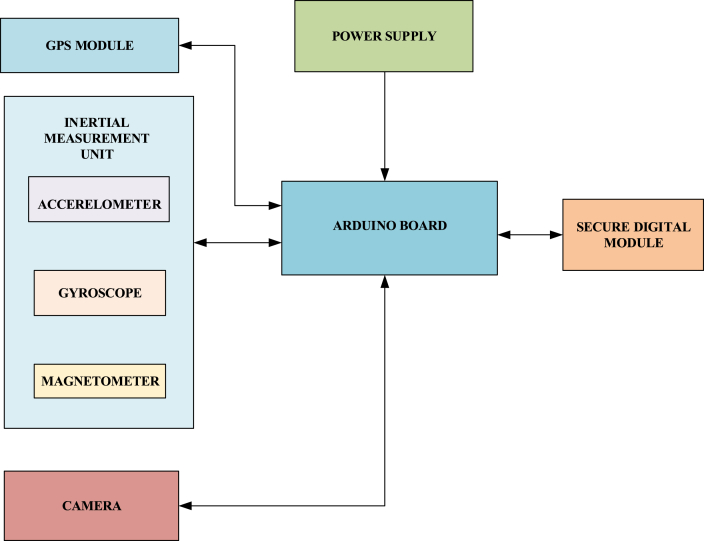
Data logging unit.

The above figure-1 shows the data logging system. A data logging system is a device that can log data from various sensors over time or concerning the location. It can either have built-in instruments or record data from external sensors. The system is small in size and operates with external battery power. The advantage of the data logger is it can be left unmonitored to collect data and later the logged information can be analyzed based on the applications.

Fig-1 in this paper consists of a low-cost Inertial Measurement Unit (IMU), a Global Positioning System (GPS) which provides positioning, navigation, and timing solutions, and a camera as an alternative sensor, particularly in challenging GPS environments. The measurements of the sensors are processed using a microcontroller and the data is recorded on an SD card. In paper [30] this was performed by using a Kalman filter. This is a traditional way of implementing the Kalman filter for sensor fusion. This type of filter requires high sampling rates for the linear regression iterations.

This paper is performed by implementing a light controller on the Arduino board. The main novelty of this practical implementation is designing a Proportional-Integral (PI) control strategy that deals with external disturbances effectively. This paper also describes the controller tuning process. Precise orientation feedback is provided by employing a measuring system based on the Mahony filter.

### 2.1 Unmanned Aerial Systems

It operates from remote locations thanks to its collection of unmanned aerial vehicles and ground control stations. An essential part of the UAV is the data logging device, which collects scientific data from all sensors [13]. The author [19] presented six challenges to using small Unmanned Aerial Systems (sUAS) for environmental remote sensing: the hostile flying environment, the power challenge, the available sensors challenge, the payload weight challenge, the data analysis challenge, and the regulatory challenge.

### 2.2 Data logging system

The procedure of gathering and storing flight data is supplied by the data acquisition unit. The recording system varies in size, power consumption, and weight. Data stored separately can also be used for analyzing processes. A collection of sensors for calculating the UAV flight parameters, software for visualizing the recorder data, and a memory card installed with a microcontroller-based flight data recorder are all included in this unit. Data logging, data acquisition, and data processing are all significant in this system [14,15].

The author [16] says that the data acquisition device offers helpful information about the three types of air data, inertial, and control displacement dynamics of aircraft. The inertial instruments comprise three-axis linear magnetometers, three-axis angular rate gyros, and three orthogonally mounted angular accelerometers.

For real-time measurement and storage of flight parameters flight data recorders (FDR) is used [17]. The roll, pitch, and yaw angles of an airplane are calculated using a triple-axis accelerometer and gyroscope [18]. The lack of a magnetometer in the sensor caused the yaw angle readings to be inaccurate; the 3-axis magnetometer fixes this kind of issue.

### 2.3 Arduino controller

The Arduino is an open-source controller used for controlling analog devices, digital devices, and electronic components. The software programming languages used for this purpose are C and C++. Arduino microcontroller is used in the paper as it has enough digital pins required for the camera. The Arduino is a free and simple-to-use piece of software.

However, the advent of low-cost microcontrollers like the Arduino has permitted the construction of low-cost smart home systems that incorporate the bulk of the functions found in commercial systems. We describe in this work a highly scalable, low-cost, and multi-faceted home automation system based on the Arduino microcontroller, capable of combining appliance and equipment automation, thermal comfort control, and energy management [20].

The Arduino is used to manage and coordinate the various sensors that are wired to it. The data collected by the sensors is also processed by the system.

And also the system covers the development and design of a weather station utilizing an Arduino board and five sensors that provide data for the sixth reading (rain state, wind level, air pressure, dust density, temperature, and humidity). When receiving data from many transmitter nodes (clouding and main processing side), the data might be saved in an SD card. The data may be accessed at any time and date. The results demonstrated that the system has no latency and that the data is supposedly updating every second with each new measurement [21].

The ability of the Arduino to connect with other hardware components using UART (hardware serial), I2C (two wires), and SPI (four wires) is one of its distinguishing features. I2C is the most user-friendly of these since it can be interfaced with the microcontroller with the fewest possible cables and because it is compatible with the Arduino Libraries. Data is typically transferred between micro-controllers and SD cards using the Serial Peripheral Interface (SPI). It accomplishes this through a data line and real-time clock. Despite the microcontroller's limitations, the user can create virtually anything with Arduino because of the platform's open-source nature and the wide availability of library code. Arduino is utilized to regulate and coordinate all of the sensors that are linked to it. It also processes the raw data collected by sensors. It is an open source arduino board equipped with magnetometers, accelerometers, and barometers to provide the essential feedback values for flying [22].

### 2.4 Inertial measurement unit (IMU)

To measure accelerations and rotational speeds along all three of your body's axes with the help of an IMU's accelerometers and gyroscopes. When compared to GPS, IMU readings are not impacted by the object's surrounding conditions. An IMU is a structure that includes an accelerometer, gyroscope, magnetometer, and barometer working together to measure the aircraft's altitude, orientation, and gravitational forces. Accelerometers measure changes in position. Gyroscopes are devices that maintain rotational motion by measuring rotational angles. Magnetometers measure magnetic fields. They are used along with a gyroscope to correct the gyroscope's drift. A barometer is used to measure altitude through pressure measurement. The accelerometer, gyroscope, and magnetometer work as units to compensate for each other's pitfalls to provide drift-free orientation. An Inertial Measurement Unit operates by measuring the rate of change of acceleration using an accelerometer and measures changes in rotational attributes (pitch, roll, and yaw) using a gyroscope and magnetometer. Also, IMU assists in the calibration of the sensors to provide error-free measurement [23].

### 2.5 Accelerometer

The accelerometer has a high resolution of about 13 bits and can measure up to + -16 g. A digital output data is in a 16-bit twos-complement encoding. It is accessible via the SPI or I2C interface. In this paper, communication is established through the I2C protocol. In tilt sensing applications, the accelerometer is used to measure static acceleration. Here, gravity is the acceleration measured. It can also be used to measure the energetic acceleration ensuing from motion or shock. Due to its high resolution (4 mg/LSB), an inclination of fewer than 1.0° can be measured. The scale factor of an accelerometer is the input-to-output relationship of acceleration changes. Standard measurements are LSB per gram or mg per LSB. The accelerometer makes this quite easy, as just a few short calculations are required. All of these procedures have to be performed after each iteration's readout of the sensors' results [24].

### 2.6 Gyroscope

It is a three-axis low-power angular rate sensor. It supports a full scale of ±250/±500/±2000 dps. It measures rates based on user-selectable bandwidth. In this paper, the gyroscope output data range is selected as 100Hz since it is the minimum frequency at which the sensor outputs the measurement. This does not affect overall IMU as the gyroscope operates at a higher frequency (0.01s) than the main loop which runs at 50Hz (0.02s). The sensor is set for continuous data update and little Endean data selection. A full scale of + -2000 dps (degrees per second) is used for the measurement of angular velocity.

### 2.7 Magnetometer

The Magnetometer is manufactured by Honeywell which can measure low magnetic variations. The device is controlled through the I2C protocol. Unfortunately, the measurements are associated with an error during the tilt of the aircraft [15]. To eliminate this error, the tilt compensation method has to be implemented. The tilt compensation method requires an accelerometer sensor (x,y). By definition, a magnetometer is an instrument used to measure and analyse magnetic fields. A UAV can determine the location of magnetic north and adjust its course indefinitely [9].

## 3 Directional cosine matrix

In this filter, the rotation of the aircraft is represented by a rotational matrix [15]. The elements of the matrix are maintained through information from the accelerometer, gyroscope, and magnetometer. This implies that the orientation of planes is shown about the axis of the Earth's rotation. The greatest fit for navigation and control is a rotational matrix. They are symbols for the relative direction of two coordinate systems. A rotation matrix specifies how one coordinate system is oriented with relation to another. The matrix's columns are the unit vectors in one system as observed in the other [26]. Integrating a nonlinear differential equation that describes the kinematics of the rotation preserves the rotation matrix that determines the orientation of the aircraft. Kinematics deals with the geometry of the rotation and rotational transformation of one configuration to another. The integration is achieved through matrix multiplications at the rate of 50Hz. Elements of the matrix are adjusted to eliminate numerical errors. Proportional and Integral controller is used for error dissipation. The basic diagram of the working of the DCM filter is shown below.

**Fig-2:**
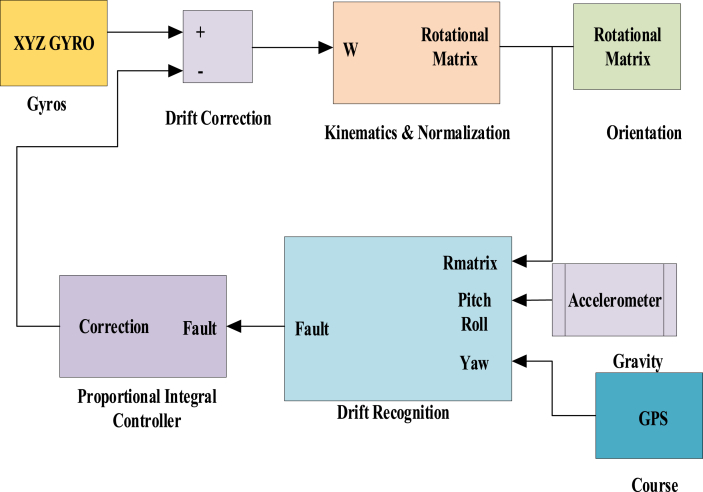
Directional Cosine Matrix block diagram.

The above figure-2 shows the Directional Cosine Matrix (DCM). It was developed so planes could progress from being inherently stable with elevator and rudder control to aerobatic planes with ailerons and elevators [25]. A fixed coordinate system should be considered to describe the aircraft's motion. In this paper, the Earth coordinate system is considered as a reference. These angular rotations are referred to as Euler angles and are used to characterize the aircraft's orientation through a series of three consecutive rotations. Instead of Euler angles or quaternions, this work presents a technique for estimating the direction cosine matrix (DCM), which includes attitude information. The DCM does not have singularities and has linear dynamics. From fig-2 the DCM estimate technique was derived, which includes automated magnetometer bias calibration and fulfilment of a DCM's intrinsic orthonormal feature [26]. These angles are yaw, pitch, and roll. They are determined with reference to the earth's synchronized system (Xg, Yg, and Zg). For this, first, the coordinates of the aircraft are made parallel to the earth's and rotated along each axis. Rotation around its Z axis (Zb) provides a yaw angle, rotation around the Yb axis gives a pitch angle, and the Xb axis gives a roll angle.

(1) $$R=\left|\begin{array}{ccc} r_{xx} & r_{xy} & r_{xz}\\ r_{yx} & r_{yy} & r_{yz}\\ r_{zx} & r_{zy} & r_{zz} \end{array}\right|$$

The rotational matrix is shown in equation (1)

The Euler angles in terms of the rotational matrix are shown below

(2) $$R=\left|\begin{array}{ccc} \cos\;\theta \;\cos\;\psi & \sin Ф\sin\;\theta \;\cos\;\psi ‐\cos Ф\sin\;\psi \; & \cos Ф\sin\;\theta \;\cos\;\psi +\sin Ф\sin\;\psi \\ \cos\;\theta \;\sin\;\psi & \sin Ф\sin\;\theta \;\sin\;\psi +\cos Ф\cos\;\psi & \cos Ф\sin\;\theta \;\sin\;\psi ‐\sin Ф\cos\;\psi \\ ‐\sin\;\theta & \sin Ф\cos\;\theta & \cos Ф\cos\;\theta \end{array}\right|$$

The Euler Angles Rotational matrix is shown in equation (2)

### 3.1 Drift cancellation in gyroscope

For the cancellation of drift in the gyroscope, orientation references from the accelerometer and magnetometer are considered to detect offsets in gyroscopic measurements. This is provided as negative feedback through the PI feedback controller to gyroscopic measurements to compensate for the error produced. This is done through the computation of the rotation vector. The rotation error is fed back into the feedback loop to determine the rotation rate adjustment the for gyroscope signal. The output of the PI controller is added or subtracted with the gyroscope signal based on sign convention.

It is possible to calculate the orientation error by getting the cross product of the measured vector and the vector in the direction cosine matrix. The magnitude and direction of the cross-product determine the axis and rotational angle to make the measured vector parallel with the estimated vector in DCM. This will be negative of orientation rotational error. In mathematical computation, cross-product is performed by exchanging the order. The cross-product determines the correction factor. These correction factors are multiplied by weights and then used in a feedback loop. The total correction will be the addition of correction factors multiplied by weights. Inputs to the PI controller are as below.

(3) ω=PCorrectionKPTotalCorrection

(4) ω=ICorrectionωKICorrection+IdtTotalCorrection

(5) ω=CorrectionωPCorrection+ωICorrection

Equation (3) shows the Proportional correction and equation (4) depicts the Integral correction values. The total correction is the sum of Proportional correction and Integral correction is shown in equation (5).

Compensated pitch, yaw, and roll values are obtained from an updated directional cosine matrix using the following formulae.

(6) pitch=‐asin(DCMMatrix[2][0])

(7) roll=atan2(DCMMatrix[2][1],DCMMatrix[2][2])

(8) yaw=atan2(DCMMatrix[1][0],DCMMatrix[0][0])

The above equations are Euler angles. Equation (6) is used to calculate the Pitch from the updated DCM. Equation (7) is used to calculate the Roll and Equation (8) for calculating Yaw from the Directional Cosine Matrix.

### 3.2 GPS module

Anywhere on or near the surface of the Earth with a clear line of sight, four or more GPS satellites can use the Global Positioning System (GPS) to determine their precise location and the exact time. The accelerometer guarantees a more accurate reconstruction of the traveled path when used in conjunction with GPS since it can detect the gyro roll-pitch drift contained within the same IMU [27]. The author in Ref. [28] most prevalent is undoubtedly a fusion with inertial measurement units. The primary goal of this work was to evaluate the kinematic positioning capabilities of a low-cost u-blox NEO-M8U module. Despite the fact that the module is primarily aimed at the automobile sector, it has been evaluated for performance in low-speed applications. GPS module is used in the paper to retrieve measurements like latitude, longitude, and timestamp.

### 3.3 Camera with FIFO memory

The next area of interest of this paper is to select a suitable low-cost camera and program it to capture pictures at every remote trigger. The method of triggering can be modified from a remote trigger to a time-based trigger through a software program. It is available in two variations: one that does not have frame buffer memory and another that does have frame buffer memory and is referred to as the FIFO variant. The suggested embedded system is built on an Omnivision OV7670 color camera and a CPU [29].

## 4 Stabilization of yaw, pitch, and roll values

The yaw, pitch, and roll values are stabilized with the calibration of sensors and the implementation of a Directional Cosine Matrix-based Mahony filter. The graphs depicting the stabilized values are shown below. The graphs are plotted using the Serial Plotter tool in Arduino IDE. The measurements were made on flat surface with minimal vibrations and low exposure to magnetic field.

**Fig-3:**
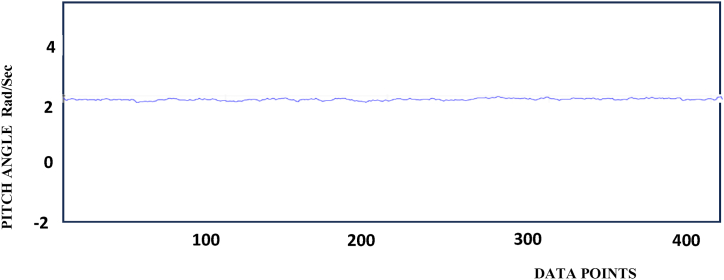
Pitch stabilization.

The Pitch values are stabilized with the calibration of sensors are shown in Figure-3.The table-1 shows the reading taken from Arduino IDE and the data are predicted for the specified pitch values.

**Table-1:** Pitch measurements.

Readings	Data Points (X-Axis)	Pitch Angle (Y- Axis) Rad/Sec
1	50	2.2
2	100	2.1
3	150	2.6
4	200	2.8
5	250	2.5
6	300	2.4
7	350	2.2
8	400	2.8

**Fig-4:**
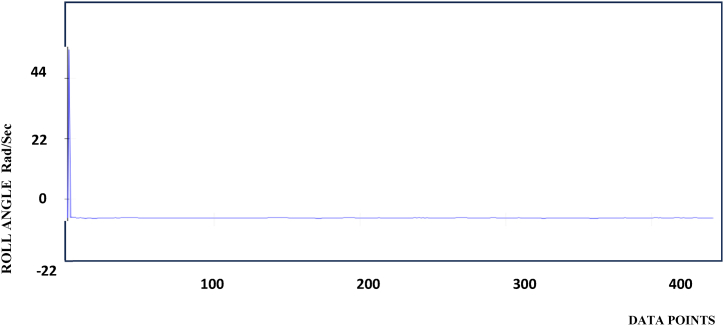
Roll stabilization.

The Roll values are stabilized with the calibration of sensors are shown in Figure-4 Table 2 shows the reading taken from Arduino IDE and the data are predicted for the specified roll values.

**Table-2:** Roll measurements.

Readings	Data Points (X-Axis)	Pitch Angle (Y- Axis) Rad/Sec
1	50	−7.2
2	100	−7.3
3	150	−7.4
4	200	−7.3
5	250	−7.2
6	300	−7.3
7	350	−7.4
8	400	−7.6

**Fig-5:**
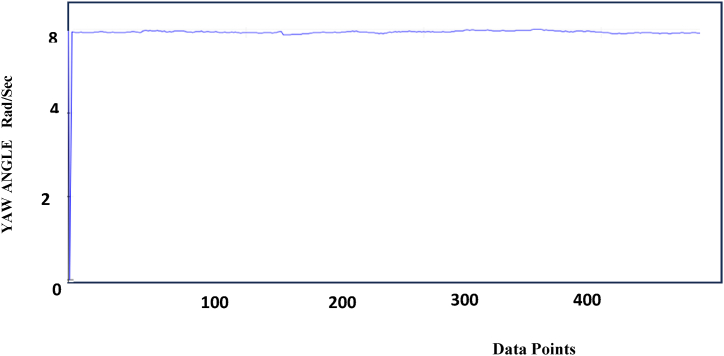
Yaw stabilization data Points data POINTS

The Yaw values are stabilized with the calibration of sensors are shown in Figure-5. Table-3 shows the reading taken from Arduino IDE and the data are predicted for the specified Yaw values.

**Table-3:** Yaw measurements.

Readings	Data Points (X-Axis)	Pitch Angle (Y- Axis) Rad/Sec
1	50	8.3
2	100	8.4
3	150	8.3
4	200	8.5
5	250	8.6
6	300	8.2
7	350	8.23
8	400	8.35

The Pitch, Roll, and Yaw axis stabilization behavior is shown in Fig-3, Fig-4, Fig-5. From the diagram, the Pitch, yaw, and roll of the device have stabilized to bring the moving object into a position, and the orientation can be calculated and the values are logged. Based on the value for stabilization provided it will show that particular values. It can be seen that the controller successfully drives the system pitch Roll and Yaw outputs to follow the reference values.

## 5 CONCLUSION and FUTURE WORK

The data logger system was successfully designed and implemented. Based on the requirements, the devices were selected and programmed. The challenges encountered due to drift in the IMU data were addressed through calibration and filter.

As a part of future enhancement, sophisticated filters can be implemented to achieve more stabilization in IMU data. The paper was developed for low-altitude applications due to the use of the camera. In the future, this can be replaced with high-resolution cameras for higher-end applications. By comparing the conventional ways of designing filters like Kalman filters this paper provides efficient and also effective ways for IMU. And also the execution time of this filter is less than the conventional methods. In the future high resolution camera can be used. The logged data can be transferred to the base station through the transmitter and receiver section for controlling and also for further analysis the current situation.

## Declarations

### Ethics approval

Not Applicable.

## Consent to participate

Not Applicable.

## Consent for publication

Not Applicable.

## Availability of DATA and materials

Data will be available based on the request.

## FUNDING

Not Applicable.

## Authors contributions

The authors confirm their contribution to the paper as follows: design and implement of Data logging in UAV, Abhishek Gudipalli, and Siva Sivamani Ganesh Kumar.

## CRediT authorship contribution statement

**Ganesh Kumar Siva Sivamani:** Writing – original draft, Visualization, Validation, Software, Resources, Methodology, Formal analysis, Data curation. **Abhishek Gudipalli:** Writing – review & editing, Validation, Supervision, Resources, Project administration, Data curation.

## Declaration of competing interest

The authors declare that they have no known competing financial interests or personal relationships that could have appeared to influence the work reported in this paper.

## LIST OF ABBREVIATIONS

UAV: Unmanned Aerial Vehicle
RPM: Revolutions Per Minute
SD: Secure Digital
PC: Personal Computer
SPI: Serial Peripheral Interface
UART: Universal Asynchronous Receiver/Transmitter
DCM: Directional Cosine Matrix
I2C: Inter-Integrated Circuit
LSB: Least Significant Bit
FIFO: First In First Out
IDE: Integrated Development Environment
GPS: Global Position System
IMU: Inertial Measurement Unit
